# Oral peripheral ameloblastoma: A retrospective series study of 25 cases

**DOI:** 10.4317/medoral.22225

**Published:** 2018-04-24

**Authors:** Xinyu Zhang, Xuerui Tian, Yongjie Hu, Chenping Zhang, Cao Wei, Xi Yang

**Affiliations:** 1 MD, PHD, Department of Oral & Maxillofacial – Head & Neck Oncology, Ninth People’s Hospital, Shanghai Jiao Tong University School of Medicine, Shanghai Key Laboratory of Stomatology, Shanghai, China

## Abstract

**Background:**

Peripheral ameloblastoma (PA) is a rare and unusual variant of odontogenic tumor, which was described only in isolated case reports in literature. The objective of this study was to investigate the clinical profile, treatment and outcome of PA in a consecutive case series.

**Material and Methods:**

A total of 25 patients with histologically confirmed PA from 2001 to 2015 were retrospectively reviewed in our institution.

**Results:**

Of the 25 patients, 22 males and 3 females were identified (male: female = 7.3:1). The average age was 48.3 years (range 11-81 years) with lingual or palate gingival region being the most common site (76%). The course of disease was less than 6 months in 92.0% (23/25) of all patients (mean, 3.3 months; range, 1-12 months). All patients underwent complete surgical removal of the lesions, and one lesion recurrence occurred during the follow-up period.

**Conclusions:**

The clinical profile and outcome of PA from Eastern China were elucidated in this retrospective analysis based on a case series. Our experience may provide some insights into the differential diagnosis and clinical management of PA. The first choice of treatment is surgical excision, which can result in a good prognosis.

** Key words:**Peripheral ameloblastoma, clinical profile, outcome.

## Introduction

Peripheral ameloblastoma (PA) is a rare and unusual variant of odontogenic tumor, which accounts for 1-5% of all ameloblastomas. It is also known as the extraosseous ameloblastoma, soft tissue ameloblastoma, ameloblastoma of mucosal origin, or ameloblastoma of the gingiva (1). Refering to definition of World Health Organization (4th edition, 2017), ameloblastomas were classified as solid/multicystic, peripheral/extraosseous, desmoplastic and unicystic types. Currently, the classification has been simplified and narrowed to ameloblastoma, unicystic ameloblastoma and peripheral/extraosseous types (2). PA is an exophytic growth localized to the soft tissues overlying the tooth-bearing areas of the jaws and does not invade the underlying bone. PA shows several histologic characteristics of an intra-osseous infiltrating ameloblastoma, but PA with histologically low-grade malignant features is extremely rare (1).

PA was first reported in the literature by Kuru in 1911 (3), and a case report by Stanley and Krogh in 1959 was considered to be the first well-established case of PA (4). Up to now, only approximately 210 cases of PA have been reported in the English-language literature (1,5-20). Hardly any consecutive case series studies on clinical profile and outcome of PA are available so far, and this disease was described only in isolated case reports in literature. Therefore, a single-institution series of patients with PA in oral cavity (n = 25) were reviewed to investigate the clinical profile, treatment and outcome in a retrospective hospital-based study from China.

## Material and Methods

All the medical records of patients with pathological diagnoses of PA (n = 25) from January 2001 to December 2015 were reviewed retrospectively in a standard computerized database from the Shanghai Ninth People’s Hospital, Shanghai Jiao Tong University School of Medicine. The histopathologic diagnosis of all cases was routinely determined by an oral pathologist on duty from the Department of Oral Pathology, Ninth People’s Hospital, Shanghai Jiao Tong University School of Medicine. According to the WHO criteria for PA(2,21), reexamination and confirmation diagnosis of PA was performed by another oral pathologist (J. Li). Information regarding gender, age, site of lesions, and clinical data was documented in detail in the records. All patients received surgical removal of the lesions with or without partial bone resection, and periodic follow-up examinations at intervals of every 6 months in the first 2 years and at least every 12 months thereafter were recommended for patients. This study was approved by the local institutional review board.

## Results

- Patient demographics

Of the 25 PA patients, 22 were males and 3 were females with a male-to-female ratio of 7.3:1. The age of the patients ranged from 11 to 81 years with a mean of 48.3 years. The majority of PA patients (48.0%) were in the fourth (n=5) and fifth (7) decade of life. There were 4 cases in the third and sixth decade of life respectively, and one case occurred in pediatric period (age <18 years).

- Course of disease 

The course of disease was less than 6 months in 92.0% (23/25) of patients (mean, 3.3 months; range, 1-12 months).

- Symptoms 

Most patients reported a gradually growing and painless mass without obvious symptoms, except for one patient reported swelling for one month and one patient reported a slightly painful mass for 2 months.

- Physical examination

The majority of lesions (76.0%) were less than 2.0 cm (mean, 1.7 cm; range, 0.5-3.2 cm). The masses were mobile, clearly palpable, and moderate to hard in hardness without obvious infiltration.

- Location 

The sites of occurrence were shown in  Table 1, and the location distribution was similar in the mandibular and maxillary regions. Most of the lesions (19/25) occurred in the lingual or palate gingival region.

**Table 1:**
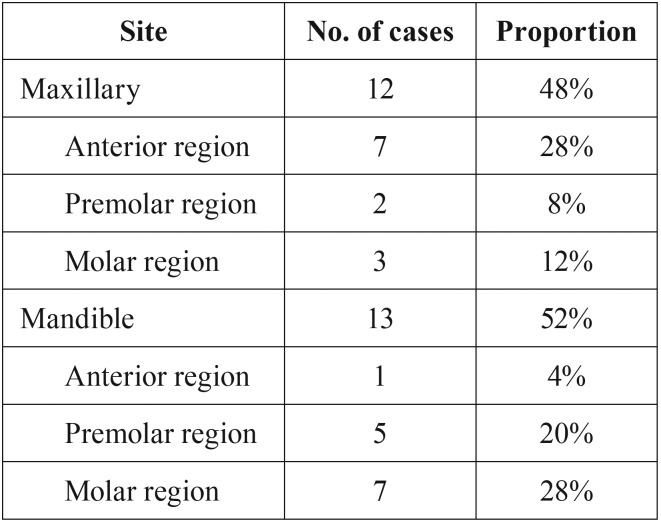
Sites of occurrence of the 25 intraoral PA cases.

- Image examination 

**Figure 1:**
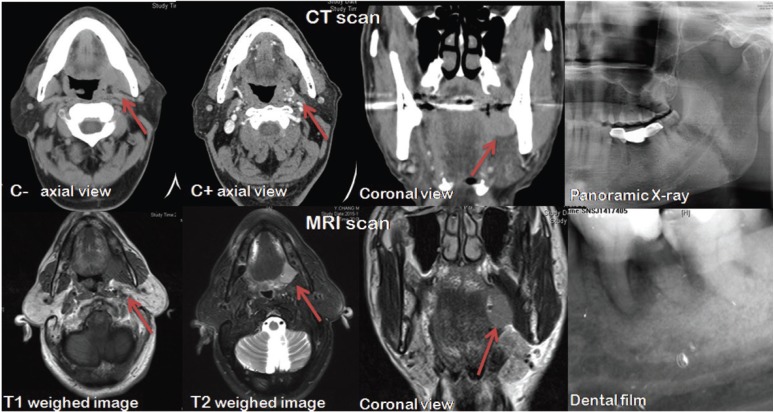
Representative image examination (CT images, MRI images, panoramic X-ray and dental film) of one case of peripheral ameloblastoma.

Computed tomography (CT) and magnetic resonance imaging (MRI) revealed a well-demarcated circular mass. However, the tumor was not enhanced on contrast CT, and a short signal was observed in T1 and T2-weighted MRI images. CT and MRI usually demonstrated no invasion to the jaws and adjacent muscles. Representative image examination of one case is shown in Figure 1.

- Laboratory examination 

Routine laboratory tests were performed in all patients, and all the results were within the normal reference ranges.

- Preoperative clinical diagnosis 

Preoperative clinical diagnosis was difficult to make, especially in the absence of biopsy or fine needle aspiration cytology. The differential diagnoses included epulis (n = 8), fibroma (n = 5), oral ulcer (n = 3), lymphoma (n = 5), and oral squamous cell carcinoma (n = 3).

- Treatment 

All patients underwent complete surgical removal of the lesions under local (1% lidocaine) or general anesthesia. All specimens were processed for routine histopathologic examination. For small lesions, conservative supra periosteal surgical excision was performed with adequate disease-free margins. While for large lesions, incisional biopsies were performed when differential diagnosis included malignant lesions before operation. Partial bone was resected if cuplike or saucerized bone involvement was detected during the operation. Primary closure and wound healing were achieved in all patients.

- Gross specimen

**Figure 2:**
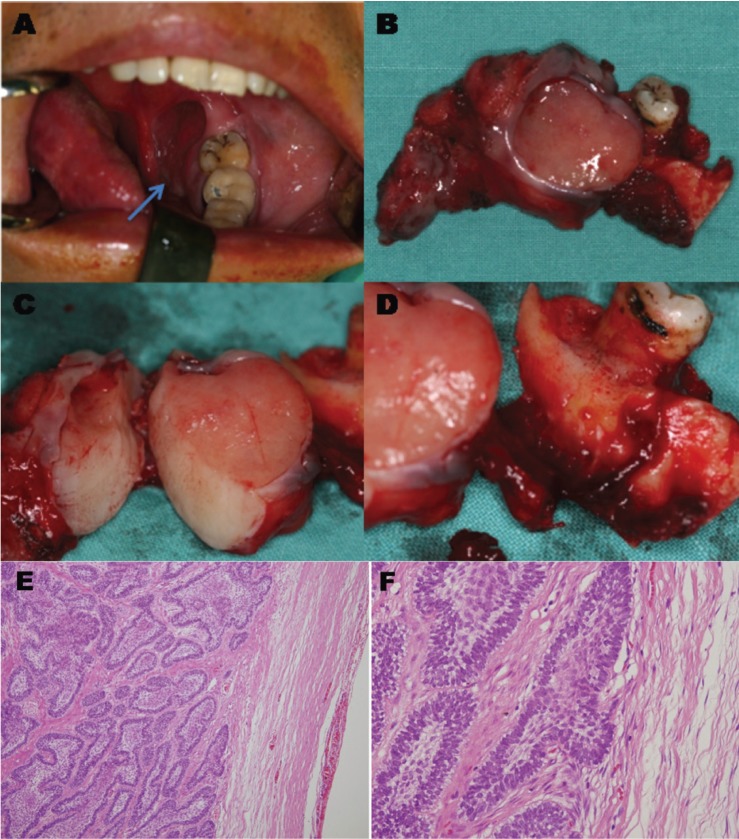
Representative clinical and pathological examination of one case of peripheral ameloblastoma (PA). (A) Front view of intraoral PA located on lingual gingiva of theretromolar region. (B) No cauliflower-like hyperplasia in the mucosa. (C) Gross specimen showed a firm to slightly spongy mass of pinkish grey. (D) No bone involvement. (E, F) Representative histopathology of this case of PA. Epithelial islands exhibited palisading of columnar basal cells and satellite reticulum was seldom conspicuous (Hematoxylin-Eosin staining). Magnification, E, ×100, F . ×200.

The lesions were mostly described as a firm to slightly spongy mass of pink to pinkish grey. The cut surface may contain minute cystic spaces filled with clear, pale-yellow fluid. As occasional areas of dystrophic calcification were very small, they were not disclosed by cutting through the specimen or on a radiograph of the operation specimen. The size of the masses ranged from 0.5*0.3cm to 3.2*3.0cm with a mean of 1.5*1.4 cm. Re-presentative gross specimen of one case is shown in Figure 2 (A-D).

- Pathologic diagnosis

The postoperative pathologic diagnosis was PA in all patients. Most of the epithelial islands exhibited palisading of columnar basal cells, and stellate reticulum was seldom conspicuous. Bone or periosteum was not involved in the pathology of PA patients. Representative histopathology of one case is shown in Figure 2 (E, F).

- Follow-up 

The follow-up period of the patients ranged from 3 to 180 months with a mean of 61 months. During the follow-up period, only one case recurred. The recurrent PA developed from the general site of the original lesion, and the reason was speculated to be incomplete removal rather than aggressiveness. Overall, all patients had good quality of life.

## Discussion

In the English-language literature, there were few case series of the demographics and clinical data of PA because of its low incidence rate. This disease was described only in isolated case reports in literature. According to the clinical data of literature review (1,4), PA occurs more frequently in males than in females, with a male-to-female ratio of 1.8:1. However, the male-to-female ratio is as high as 7.3:1 with an obvious male-predominant in our case series. This was probably due to the ethnic population and geographic difference. PA can occur at all ages (rang, 9-92 years) but most frequently in adults aged 40 to 60 years. It is very rare in children, and the earliest age of occurrence was repor-ted in a 9-year-old male (1). In our series of 25 patients, the majority of PA patients (64.0%) were in the fourth to sixth decade of life (range, 11-81 years), with a mean age of 48.3 years. The youngest patient in our series was an 11-year-old female with the lesion in the left anterior maxillary region.

PA is frequently an incidental finding during a routine dental examination. As such, it is a challenge for clinicians to make a correct diagnosis at its first presentation. Imaging modalities, such as CT and MRI, may be helpful for the diagnosis as they can sometimes clearly demarcate the lesions. This is because in most cases, the lesions are located near the bone and within the normal tissue boundaries. Bone involvement known as cupping or saucerization is rare in PA patients. Saucerization refers to a depression made from the pressure of the tumor on bone. However, it is usually mild with no neoplastic invasion or marrow infiltration (1). The dense fibrous tissue of the gingiva and periosteum and the cortical plate of the alveolar process may be responsible for an effective barrier to the infiltration of PA. The biological behavior of PA is consistent with that of a hamartoma or persistent hyperplasia rather than that of a neoplasm.

The clinical manifestations of PA, such as the course of disease, lesion growth and symptoms, are not specific for PA, making it difficult to distinguish between PA and some other lesions, such as epulis, fibroma, squamous cell carcinoma, and lymphoma (1,13). For intraoral lesions, ultrasonic examination was rarely performed, and the lesions could be incorrectly diagnosed as epulis or periapical fistula. Based on a relatively large number of case series, some experience was summarized as following. The diagnosis of PA may be considered if: (i) the mass grows slowly without pain and trismus;(ii) no cauliflower-like change in the superficial mucosa or less mucosal lesion than submucosal mass; and (iii) CT or MRI shows clear boundary between bone and medialpterygoid muscle, uniform density and less enhanced images. In these cases, fine needle aspiration or biopsy is strongly recommended to prevent unnecessary surgical intervention.

For small lesions, conservative supra periosteal surgical excision with an adequate disease-free margin is recommended even in the case of no confirmed diagnosis. While for large lesions, incisional biopsies were performed when differential diagnosis included malignant lesions before operation. Partial bone was resected if cuplike or saucerized bone involvement was detected during the operation. Continuous follow up is necessary due to the possibility of late recurrence or malignant changes.

In summary, the clinical profile and outcome of PA from Eastern China were elucidated in this retrospective analysis based on a case series. Our experience may provide some insights into the differential diagnosis and clinical management of PA. The first choice of treatment is surgical excision, which can result in a good prognosis.
